# Spike Protein Mutation-Induced Changes in the Kinetic and Thermodynamic Behavior of Its Receptor Binding Domains Explain Their Higher Propensity to Attain Open States in SARS-CoV-2 Variants of Concern

**DOI:** 10.1021/acscentsci.3c00810

**Published:** 2023-09-21

**Authors:** Jasdeep Singh, Shubham Vashishtha, Bishwajit Kundu

**Affiliations:** † Department of Chemistry and Biochemistry, University of Denver, Denver, Colorado 80208, United States; ‡ Kusuma School of Biological Sciences, Indian Institute of Technology-Delhi, New Delhi 110016, India

## Abstract

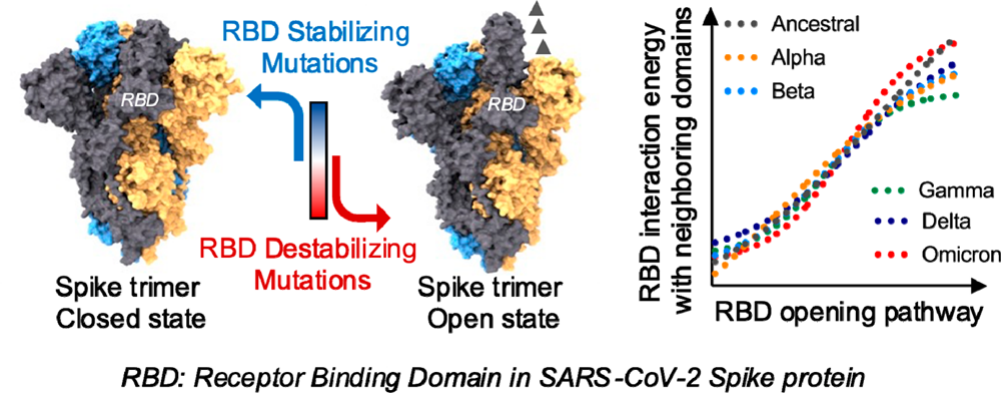

Spike (S) protein opening in SARS-CoV-2 controls the accessibility of its receptor binding domains (RBDs) to host receptors and immune recognition. Along the evolution of SARS-CoV-2 to its variants of concern (VOC)—alpha, beta, gamma, delta, and omicron—their S proteins showed a higher propensity to attain open states. Deciphering how mutations in S protein can shape its conformational dynamics will contribute to the understanding of viral host tropism. Here using microsecond-scale multiple molecular dynamics simulations (MDS), we provide insights into the kinetic and thermodynamic contributions of these mutations to RBD opening pathways in S proteins of SARS-CoV-2 VOCs. Mutational effects were analyzed using atomistic (i) equilibrium MDS of closed and open states of S proteins and (ii) nonequilibrium MDS for closed-to-open transitions. In MDS of closed or open states, RBDs in S proteins of VOCs showed lower thermodynamic stability with higher kinetic fluctuations, compared to S proteins of ancestral SARS-CoV-2. For closed-to-open transitions in S proteins of VOCs, we observed apparently faster RBD opening with a 1.5–2-fold decrease in the thermodynamic free-energy barrier (Δ*G*_closed→open_). Saturation mutagenesis studies highlighted S protein mutations that may control its conformational dynamics and presentation to host receptors.

## Abstract 2

Mutations at the interaction interface of receptor binding domains modulate the kinetic and thermodynamic behavior of its conformational transitions in spike proteins of SARS-CoV-2 variants of concern.

## 1 Background

**Figure 1:**
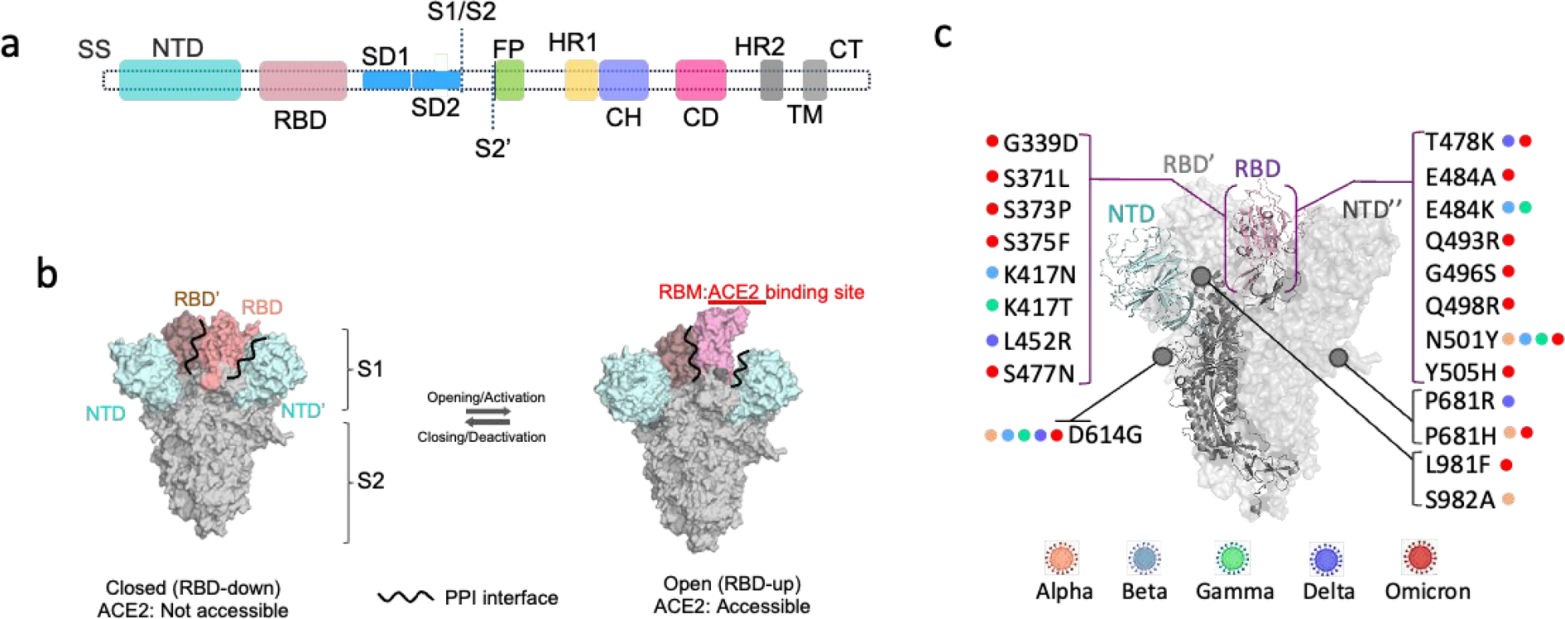
Structural and mechanical features of the SARS-CoV-2 S protein. (a) Organization of the SARS-CoV-2 S protein depicting S1 and S2 subunits. Domains in S1 and S2 subunits are shown in different colors. Single sequence (SS), N-terminal domain (NTD), receptor binding domain (RBD), subdomain 1 (SD1), subdomain (SD2), S1/S2 protease cleavage site (S1/S2), S2′ protease cleavage site (S2′), fusion peptide (FP), heptad repeat 1 (HR1), central helix (CH), connector domain (CD), HR2, heptad repeat 2 (HR2), transmembrane domain (TM), and cytoplasmic tail (CT). Figure adapted and image credits from Wang, M. Y., et al.,^2^ 2020, copyright Wang, Zhao, Gao, Gao, Wang, and Cao. (b) Opening transition in S protein where the RBD (pink) in the S1 subunit undegoes conformational rearrangemnt to interact with host ACE2 receptors via its receptor binding motif (RBM). (c) Structural location of highly conserved mutations within S protein of SARS-CoV-2 VOCs: alpha, beta, gamma, delta, and omicron (BA.1).

The SARS-CoV-2 S protein exists as a homotrimer on a viral surface, with each protomer/monomeric unit comprising S1 and S2 subunits.^1,2^ The S1 harbors the N-terminus (NTD) and the receptor binding domain (RBD), which act as a host receptor recognition subunit (Figure 1a).^2^ The S2 acts as a host cell membrane fusion subunit and contains a fusion peptide, heptad repeat 1, central helix, connector domain, heptad repeat 2, transmembrane domain, and cytoplasmic tail (Figure 1a).^2^ To establish initial contact with host receptors, the RBD(s) undergo large-scale structural rearrangement (opening transition) from a closed (non-ACE2-accessible) to an open (ACE2-accessible) state (Figure 1b).^1−4^ In the closed state of the trimeric S protein, the RBD of one protomer forms intermolecular polar and nonpolar protein–protein interaction (PPI) contacts with S1 and S2 subunits of two neighboring protomers.^5^ Thus, a canonical RBD opening transition would be accompanied by the disruption of its closed-state PPI networks.^3^ The opening transitions may occur stochastically^6,7^ or in the presence of host ACE2 receptors and RBD-targeting monoclonal antibodies.^8^ Interestingly, recent studies have shown that the opening transitions govern the engagement of full-length S protein with host ACE2 receptors or antibodies, which is distinct from the binding behavior of isolated RBDs.^4,5,9^ Correspondingly, S protein mutations which occur at the PPI interface of RBD and neighboring S1 and S2 subunits can modulate their PPI affinity and thus their opening behavior (Figure 1c). The evolution from ancestral SARS-CoV-2 to its VOCs [alpha (B.1.1.7), gamma (P.1), delta (B.1.617.2/AY.*), beta (B.1.351), and omicron (B.1.1.529.*/BA.*)] coincided with a higher proportion of their S proteins to remain in open states (Figure S1).^4,10−13^ This higher propensity to attain open states in VOCs could be attributed to mutations which occur at the PPI interface of RBDs and their neighboring domains. Mechanically, mutations which increase or decrease the PPI affinity could favor more closed or open states of S protein, respectively. For example, in closely related S proteins of SARS-CoV-1 and SARS-CoV-2, a few amino acid variations at the PPI interface of RBD-S1, S2 subunits culminate in a relatively easier S opening transition in SARS-CoV-1 and a higher proportion of RBDs to remain open state.^1,5,14,15^ Although the role of SARS-CoV-1 and ancestral SARS-CoV-2 S protein conformational transitions in ACE2 binding or antibody neutralization had been documented previously,^5,9,15,16^ knowledge of the contribution of mutations in modulating opening pathways in S proteins of VOCs is limited.

In the current study, using multiple MDS, we have assessed the role of S protein mutations in modulating the opening behavior of constituent RBDs. We have used a rigorous screening criteria using reported cryo-EM structures (*n* = 40) of closed-state S proteins of ancestral SARS-CoV-2 to establish the correct control for comparative analyses and to ascertain the initial effects of mutations on the PPI affinity of RBDs with their neighboring domains. We next performed (i) equilibrium (atomistic) MDS of closed and open states and (ii) nonequilibrium (closed to open) MDS of S proteins trimers of ancestral SARS-CoV-2 and its VOCs: alpha, beta, gamma, delta, and omicron (BA.1). MDS outcomes show that mutations can alter the PPI network of RBDs in the closed states and thus affect S protein opening in VOCs. Furthermore, we highlight differences in the RBD-opening pathway transitions of ancestral SARS-CoV-2 and its VOCs. Unique to the current study are differences in thermodynamic and kinetic landscapes of RBD(s) in S proteins of ancestral SARS-CoV-2 and its VOCs, in their closed-, open-state architecture or during opening transitions. Lastly, using saturation mutagenesis, we highlight S protein mutations which could modulate the PPI affinity of RBDs and thus affect their conformational dynamics. Understanding differences in activation/opening pathways will be central to the rational design of new molecules, which target druggable pockets in S protein, to lock it in the closed conformation as a therapeutic strategy.^17^ The results present thermodynamic and kinetic treatment of RBD conformational transitions, which would help in predicting the impact of future mutations on the ability of S protein for efficient host receptor scanning, ACE2 binding, molecular targeting, and transmission behavior.

## 2 Results

### 2.1 Selection of Initial S Protein Structures of SARS-CoV-2

**Figure 2:**
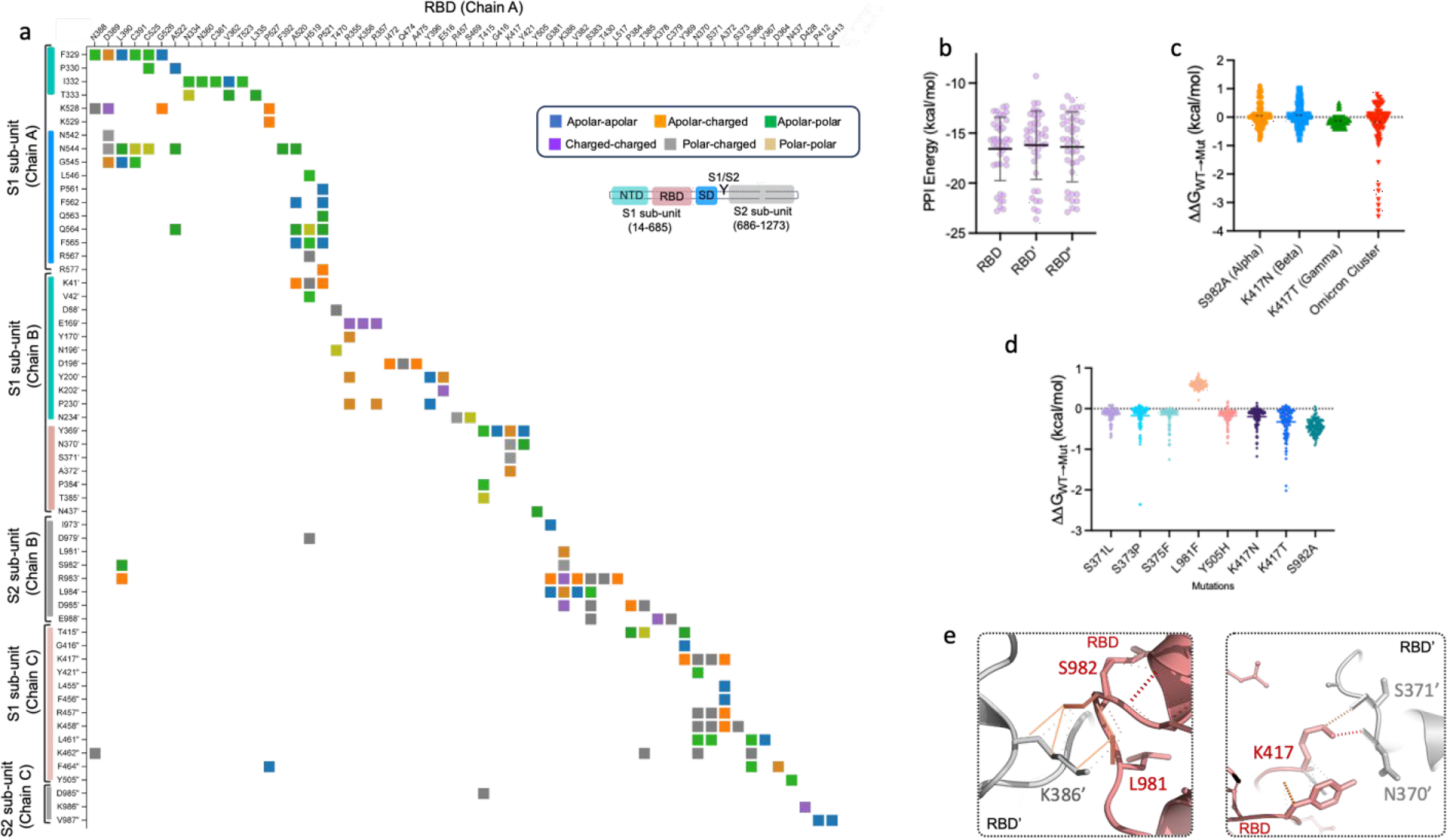
Protein–protein interaction network of spike protein RBDs in closed state. (a) Inter-residue contact map formed by single RBD in one protomer (chain A) with S1, S2 subunits/domains of the two neighboring protomers (chains B and C) in closed-state trimeric S protein. Top inset shows the type of inter-residue contacts formed of polar residues C, H, N, Q, S, T, and W; apolar residues A, F, G, I, L, V, M, P, and Y; and charged residues E, D, K, and R. Bottom inset shows S protein domain organization to depict RBD contacts with constitutent domains in neighboring S1, S2 subunits. Unannotated residues represent interdomain (RBD-NTD and RBD-SD) regions. (b) PPI energy analysis of RBDs with neighboring S1, S2 subunits in S protein trimers reported by various studies (listed in Table S1). (c, d) Changes in PPI affinity of RBDs upon acquiring various mutations occurring only at their PPI interface, calculated by FoldX-prodigy and mCSM-PPI2 methods, respectively. (e) Polar H-bond interaction network formed by interface residues between RBD and neighboring protomers, where highly conserved mutations were observed in S proteins of SARS-CoV-2 VOCs: S982 (alpha), L981 (omicron), K417 (beta, gamma, and omicron), and S371 (omicron).

In a typical closed-state S protein trimer, the RBD of one protomer (chain 1) rests within a cavity formed of S1 and S2 subunits of two neighboring protomers (chain 2: RBD′, NTD′, S2′ subunits and chain 3: RBD′′, NTD′′, S2′′ subunits) (Figure 1b,c). Thus, the resting/closed state of an RBD could be defined by its PPI network with subdomains in the same protomer and S1, S2 subunits in neighboring protomers. We first performed quantitative and qualitative analyses of the PPI network of RBD(s) in closed-state S protein using the prodigy method.^18^ Here in the PPI network, two amino acids were considered to form a contact if any of their atoms lie within a cutoff distance of 5.5 Å.^18^ The participating residues were classified as apolar (A, F, G, I, L, V, M, P, and Y), charged (E, D, K, and R), or polar (C, H, N, Q, S, T, and W), thus forming six different classes of interfacial contacts: apolar–apolar, apolar–charged, apolar–polar, charged–charged, charged–polar, and polar–polar.^18^ We observed that RBD residues (R355, N370, S371, A372, A520, D389, K386, K417, S383, and T415 in one protomer (chain A)) could form the largest and most diverse PPI network with different residues in S1 and S2 subunits of two other protomers (chains B and C) (Figure 2a). Residues Y369′, K417″, K458″, L461″, K462″, R983′, L984′, and D985′ in the neighboring S1, S2 subunits (chains B and C) could form multiple contacts with the other RBD (chain A) in its closed state. However, during the evolution of SARS-CoV-2, only selected mutations S982A (alpha), K417N (beta), K417T (gamma), and S371L, S373P, S375F, Y505H, and L981F (omicron, BA.1) were observed to be highly frequent (occurring in more than 90% of sequenced isolates) while mutations of other residues at PPI interface had a prevalence of less than 1%. Multiple sequence alignment of S protein sequences from ancestral SARS-CoV-2 and its VOCs showed that PPI interface residues were fully conserved within the NTD whereas amino acid variations were observed in the RBD and S2 subunit regions (Figure S2). We hypothesized that these interface mutations could modulate the RBD opening in S proteins of SARS-CoV-2 VOCs compared to that of the ancestral strain. Thus, the selection of the correct S protein structure of ancestral SARS-CoV-2 was necessary to avoid any bias in comparative analyses with the VOCs.

To obtain reliable control for ancestral SARS-CoV-2, we first analyzed 40 cryo-EM models of their closed-state S proteins, reported in different studies (Table S1). The missing loop regions in these models were constructed using a respective structural-template-based modeling approach, implemented in SwissModel.^19^ We then compared PPI energies of their RBDs (three per S trimer) with neighboring S1 and S2 subunits based upon a protein–protein contact-based protocol (Prodigy).^18^ This method estimates binding free energies based upon contributions from protein–protein interacting and noninteracting interfaces and does not overestimate free-energy calculations. Our analyses of these RBDs in S proteins showed a wide distribution of their PPI energies (range: −10.2 to −23.1 kcal/mol) (Figure 2b). This wide distribution of PPI energies was mainly attributed to differences in intermolecular charged and polar contacts formed by RBDs with neighboring S1, S2 subunits (Figure S3). We then analyzed S protein mutation-induced changes in PPI energies of RBDs with neighboring domains using two methods: (i) FoldX^20^-prodigy^18^ and (ii) a graph signature-based method, mCSM-PPI2^21^ (detailed in Methodology). The FoldX-prodigy method indicated a change in RBD(s) PPI energy by ±1 kcal/mol in alpha, beta and more than −3 kcal/mol in omicron (BA.1) (Figure 2c). The mCSM-PPI2 method also showed that these interface mutations, except L981F, could increase the PPI energy of RBDs (Figure 2d). Mutation-induced changes in PPI energies of RBDs could be predominantly attributed to the modulation of local polar interaction networks in S982–K386 at RBD-S2′ (alpha), K417N/T-N370 and S371 at RBD-RBD′ (beta/gamma), and L981 and K386 at the RBD-S2′ (omicron) interaction interfaces (Figure 2e). These observations indicated that the random selection of ancestral SARS-CoV-2 S protein structure could bias our comparative analyses with VOCs.

Based upon our PPI energy analyses of RBDs, we selected S protein cryo-EM structures of ancestral SARS-CoV-2 and its VOCs where the PPI energy of RBDs in their closed state varied by less than ±1 kcal/mol. To maintain consistency in our comparative analyses, we selected the cryo-EM structures of closed- and open-state S proteins, which were determined under a similar set of experimental conditions. The templates were selected using respective closed-/open-state S protein cryo-EM structures of ancestral SARS-CoV-2 (PDB id: 6vxx/6vyb)^22^ and its VOCs: alpha (PDB id: 7lws/7lwt),^11^ beta (PDB id: 7lyl/7lyn),^11^ gamma (PDB id: 8dlo/7v79),^10,23^ delta (PDB id: 7sbk/7sbl),^13^ and omicron (PDB id: 7tgy/7tgw)).^12^

### 2.2 Equilibrium MDS of Closed-State S Protein Trimers

**Figure 3:**
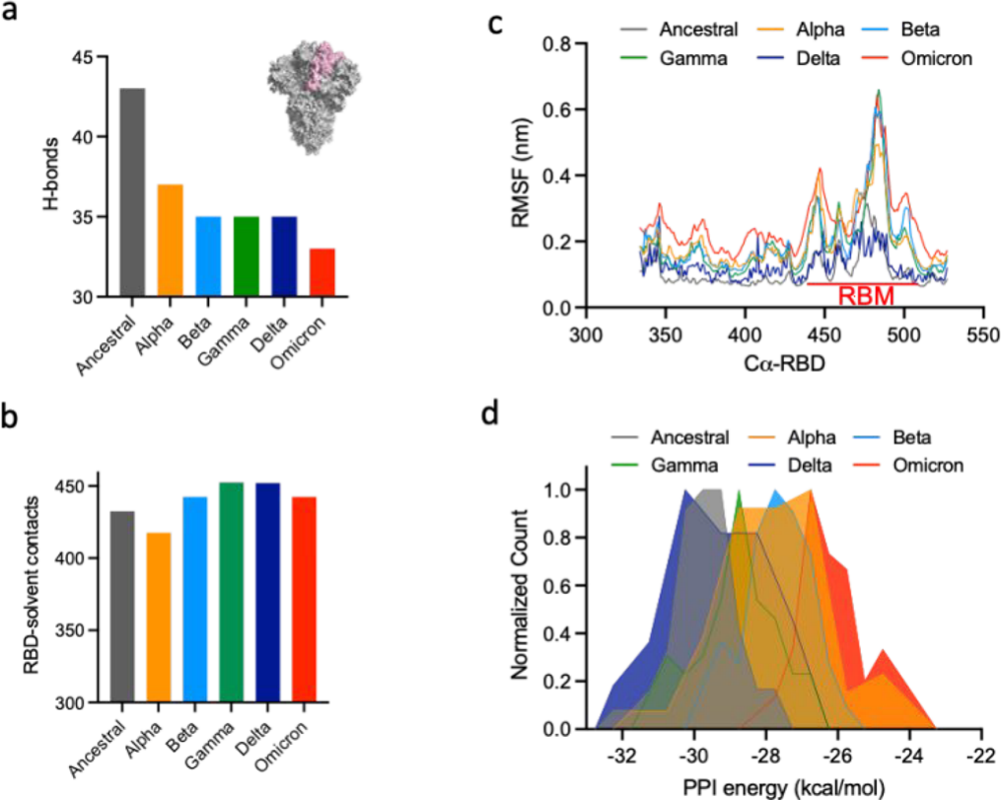
MDS of closed-state spike trimers. (a, b) Bar graphs depicting the number of H-bonds (with neigboring S1 and S2 subunits) and solvent contacts of RBD, which had the highest frequency count during simulations of closed-state S proteins of ancestral SARS-CoV-2 and its VOCs. (c) Root mean square fluctuations in Cα atoms of RBDs in ancestral SARS-CoV-2 and its VOCs, averaged over the last 20 ns of the simulation trajectory. (d) Normalized count of average PPI energy (kcal/mol) of RBDs with neighboring S1 and S2 subunits, obtained from the simulations of closed-state S proteins. Color codes for S proteins of anscetral SARS-CoV-2, gray; alpha, orange; beta, light blue; gamma, green; delta, navy blue; and omicron, red.

To understand the initial propensity of RBDs (ancestral: ^RBD^ancestral; alpha: ^RBD^alpha; beta: ^RBD^beta; gamma: ^RBD^gamma; delta: ^RBD^delta; and ^RBD^omicron) to undergo an opening transition, we performed atomistic MDS of closed-state S protein trimers. Because the RBD(s) would need to disrupt its closed-state PPI network toward the initial step for its opening transition, we first calculated the intrinsic backbone dynamics of full-length S proteins (Cα-RMSD and Cα-RMSD of RBDs with respect to the whole S protein) followed by analyses of intermolecular polar interactions of RBDs within S protein, contact frequency of RBDs with the external solvent environment, and PPI energies of RBDs with neighboring domains during the simulation period. MDS outcomes showed slightly higher Cα-RMSD for full-length S proteins of VOCs compared with ancestral SARS-CoV-2 over the simulation period (Figure S4a). Interestingly, Cα-RMSD variations of each RBD calculated through aligning to the entire spike structure presented striking differences among different variants. In two of the three RBDs in S protein, we observed a higher deviation (by ∼0.2 nm) from the starting structure in VOCs compared to ancestral SARS-CoV-2 (Figure S4b–d). In comparative distribution of polar interaction networks in S proteins, we observed that ^RBDs^ancestral formed a larger number of H-bonds (∼45) with their neighboring S1, S2 subunits whereas RBDs of VOCs formed less than 40 H-bonds (Figure 3a). ^RBDs^Omicron formed a relatively smaller number of H-bonds (∼30 H-bonds) with their neighboring domains. During the simulation period, ^RBD^beta, ^RBD^gamma, ^RBD^delta, and ^RBD^omicron were also slightly more exposed to external solvent (nearly 450 contacts) compared to ^RBD^ancestral and ^RBD^alpha (415–425 contacts) (Figure 3b). Analysis of atomic fluctuations during the late stages of simulations (last 20 ns) showed the overall lowest Cα RMSF (root-mean-square fluctuations) for ^RBDs^ancestral and ^RBDs^delta compared to other VOCs (Figure 3c). The Cα fluctuations were observed to be highest in the unstructured RBM region of ^RBD^alpha, ^RBD^beta, ^RBD^gamma, and ^RBD^omicron. We further analyzed the PPI energy of RBDs with the neighboring domains (averaged over the whole simulation trajectory) using the prodigy method.^18^ Corresponding frequency distribution plots showed the lowest (∼−30 kcal/mol) PPI energy for ^RBDs^ancestral with their adjacent domains (Figure 3d). However, compared to ^RBDs^ancestral, RBDs in S proteins of SARS-CoV-2 VOCs showed relatively higher PPI energy. While ^RBD^beta and ^RBD^gamma showed single peaks corresponding to PPI energies of −27.5 and −28.8 kcal/mol, ^RBD^alpha and ^RBD^delta displayed bimodal distributions of PPI energy [(−28.3, −26.8 kcal/mol) and (−30.2, −27.8 kcal/mol) respectively] (Figure 3d). Compared to ancestral and other VOCs, ^RBDs^omicron displayed the highest PPI energy (−26.8 kcal/mol) with the neighboring domains. MDS outcomes and protein–protein interaction analyses of RBDs from equilibrium closed-state atomistic simulations indicated slightly favorable initial states for the stochastic opening of RBDs of VOCs (more pronounced in omicron) than ancestral SARS-CoV-2.

### 2.3 Nonequilibrium Opening of S Proteins of Ancestral SARS-CoV-2 and Its VOCs

**Figure 4:**
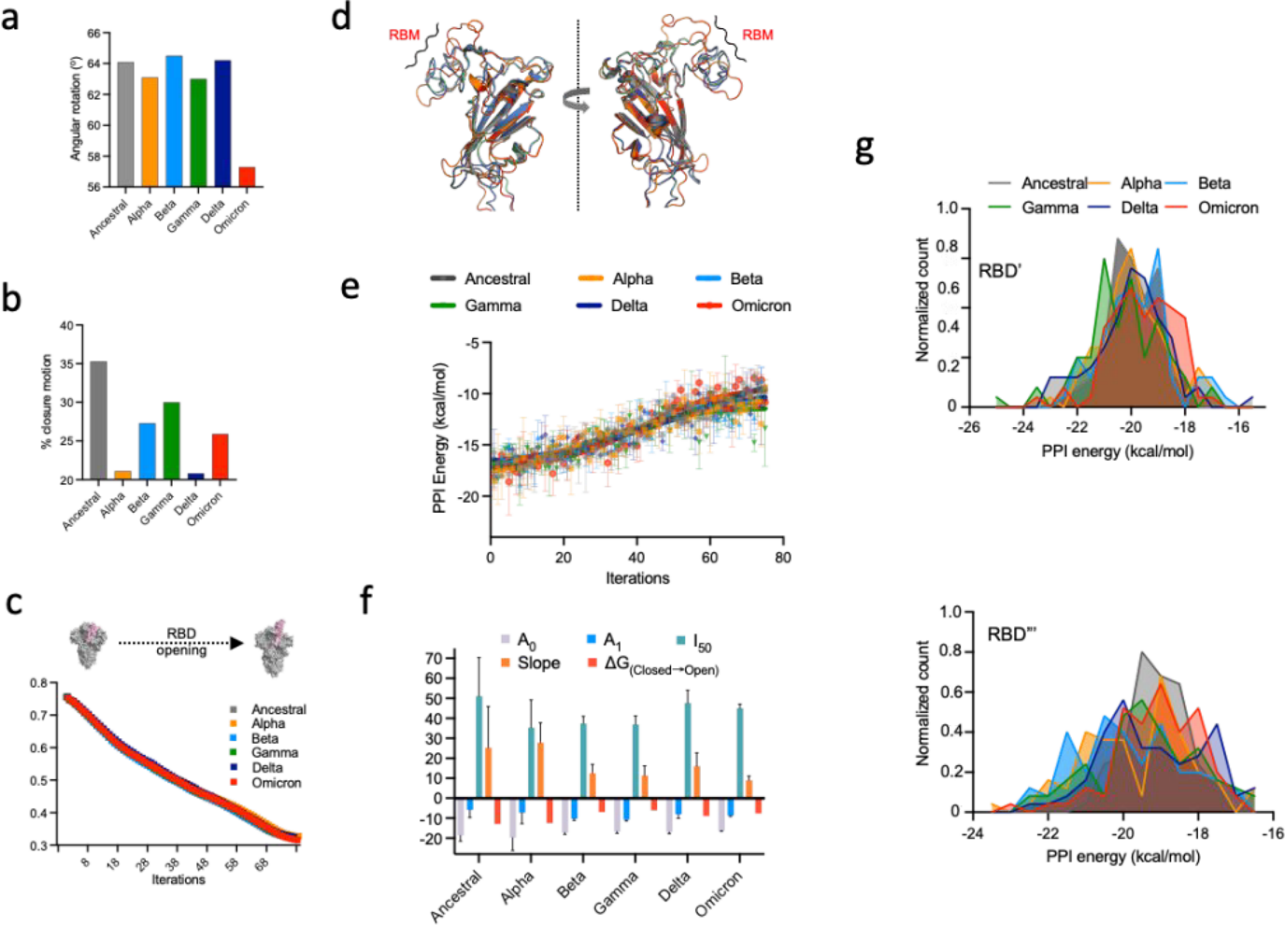
Conformational transition pathway mapping for S proteins of ancestral SARS-CoV-2 and its VOCs. (a, b) Angular rotation (deg) and closure motion (%) undergone by RBD upon rigid body domain movement from the closed to the open state in S proteins, respectively. (c) Morphing transitions indicating a decrease in Cα-RMSD for closed to open transition (within an RMSD cutoff limit of 0.3 nm) in S protein trimers. (d) Structural aligment of open-state RBDs at the end of morphing runs (red) and respective cryo-EM models (grey). (e) Variations in the PPI energy of a single RBD with its adjacent domains along its opening pathway. Solid lines depict nonlinear curve fitting of RBD PPI energy along its opening pathway. Bars indicate standard error of the mean PPI energy from two independent simulations. (f) Kinetic and thermodynamic parameters obtained from nonlinear curve-fitting analysis of the PPI energy of RBDs along the respective opening pathways. (g) Normalized distributions of the PPI energy of resting/closed-state RBD′ (chain B, top panel) and RBD′′ (chain C, bottom panel), when single RBD (in chain A) was undergoing an opening transition. Color codes for S proteins of ancestral SARS-CoV-2, gray; alpha, orange; beta, light blue; gamma, green; delta, navy blue; and omicron, red.

To determine the physical characteristics of domain movement along an opening transition, we first performed rigid body RBD movement analyses (in terms of angular rotation and closure motion) using full-length structures of closed- and open-state S proteins of ancestral SARS-CoV-2 and its VOCs. Closure motion in a conformational transition refers to domain bending movement along a closure axis, which is perpendicular to the line joining the centers of mass of fixed and moving domains. S protein opening/closing motion in ancestral SARS-CoV-2 was accompanied by 64.1° RBD angular rotation and 35.3% closure motion (Figure 4a,b). For SARS-CoV-2 VOCs alpha, gamma, and omicron, we observed a decrease in angular rotation (<64°) of RBD for an opening/closing transition. While the required angular rotation was slightly higher for beta (64.5°) and delta (64.2°) VOCs (Figure 4a), the closure motion was observed to be less than 30% for all VOCs (Figure 4b).

The conformational transition pathway for a single RBD opening in S trimers of ancestral SARS-CoV-2 and its VOCs was mapped using a composite nonequilibrium normal-mode analysis in the internal coordinates (morphing) coupled with equilibrium atomistic MDS. Using closed- and open-state S trimers as reference structures, the single-RBD opening pathway was mapped until the backbone RMSD converged to ≤0.3 nm with respect to the open state (Figure 4c). To avoid any bias in opening transitions, cryo-EM-based modeled full-length structures were used instead of previously simulated S protein trimers. The opening transition in ancestral SARS-CoV-2 and the VOCs was achieved in ∼75 iterations (Figure 4c). From the structural alignment of RBD(s) (between the open state of the reference cryo-EM model and our final structure), we observed that the backbone RMSD of ∼0.3 nm was substantially contributed by the unstructured RBM region of RBDs (Figure 4d). Subsequently, we performed short equilibrium (1 ns in duplicates) MDS of structures extracted from each iteration. We first monitored the opening transitions in terms of changes in solvent contacts of transitioning RBD and the radius of gyration (*R*_g_) of S protein trimer. The increase in the *R*_g_ of S protein timers indicated the RBD opening along the conformational transition pathway (Figure S5). Scatter plots of *R*_g_ and RBD-solvent contacts did not present significant differences among single-RBD opening transitions in S proteins. The S protein opening transition of ancestral SARS-CoV-2 showed a diffused landscape where solvent contacts of transitioning RBD remained invariable during all stages of the transition (450–550 contacts) (Figure S5). The difference in *R*_g_ between closed and open states was observed to be ∼0.17 nm. For SARS-CoV-2 VOCs, the transitioning RBD was less solvent-exposed (475–550 solvent contacts) during the initial and middle stages while the solvent contacts for a few distributions were more than 550 during the late stages of the opening transition. We further ascertained the energetics of the RBD opening (by calculating the PPI energy with neighboring domains) along the transition pathway using the contact-based prodigy method. In the one-dimensional free-energy landscapes, a single RBD opening in S protein of ancestral SARS-CoV-2 and its VOCs followed an uphill sigmoidal pathway (Figures 4e and S6). The apparent kinetic and thermodynamic parameters (A_o_: bottom of the fitted curve; A_1_: top of the fitted curve; I_50_: midpoint of iterations between A_o_ and A_1_; and S: slope of the fitted curve) derived from nonlinear curve (modified Boltzmann) fitting analysis yielded significant information and differences among the opening transitions of SARS-CoV-2 variants (Figure 4e,f). The Boltzmann equation has been previously applied to proteins which can undergo conformational changes (activation/deactivation), similar to the opening transition of the SARS-CoV-2 S protein.^24^ A_o_, which represents the PPI energy of the transitioning RBD in the closed state (at iteration = 0), was observed to be lowest for ancestral SARS-CoV-2 (−18.6 ± 2.8 kcal/mol) and higher for SARS-CoV-2 VOCs. This was also in agreement with comparative PPI energy analyses of RBDs from closed-state S protein equilibrium simulations (Figure 4f). I_50_, which represents the midpoint of a transition (where S protein has undergone half of the opening transition), indicates the apparent kinetic behavior of RBD opening transitions. The observed I_50_ for ^RBD^ancestral was 51.05 while it ranged between 35.3 and 47.6 for RBD for SARS-CoV-2 VOCs (Figure 3e). The lower number of iterations indicates faster opening transitions in S proteins of SARS-CoV-2 VOCs. Similarly, the slope of the RBD opening transition was observed to be highest (more steep) for ancestral (25.3) and alpha (27.8) SARS-CoV-2 while it was reduced by almost half (<13) for other VOCs (Figure 4f). The lowest slope value (least steep) for ^RBD^omicron indicated a flattened free-energy surface for its opening transition. To predict the propensity of S proteins to attain two or three RBD-up (open) state conformations, we further monitored changes in the PPI energy of RBD′ and RBD′′ of two other protomers, which remained in the closed state while single RBD was undergoing an opening transition. Normalized count distributions averaged over the whole reaction coordinate indicated the PPI energy, ranging between −16 and −25 kcal/mol of RBD′ and RBD′′ with the neighboring domains (Figure 4g). While we observed the highest distribution of PPI energy for RBD′ and RBD′′ of around −20 ± 1 kcal/mol, ∼40% of the population distribution (RBD′ and RBD′′) in omicron showed a slightly higher (∼−18.5 kcal/mol) PPI energy than other variants.

### 2.4 Equilibrium MDS of Open-State S Protein Trimers

**Figure 5:**
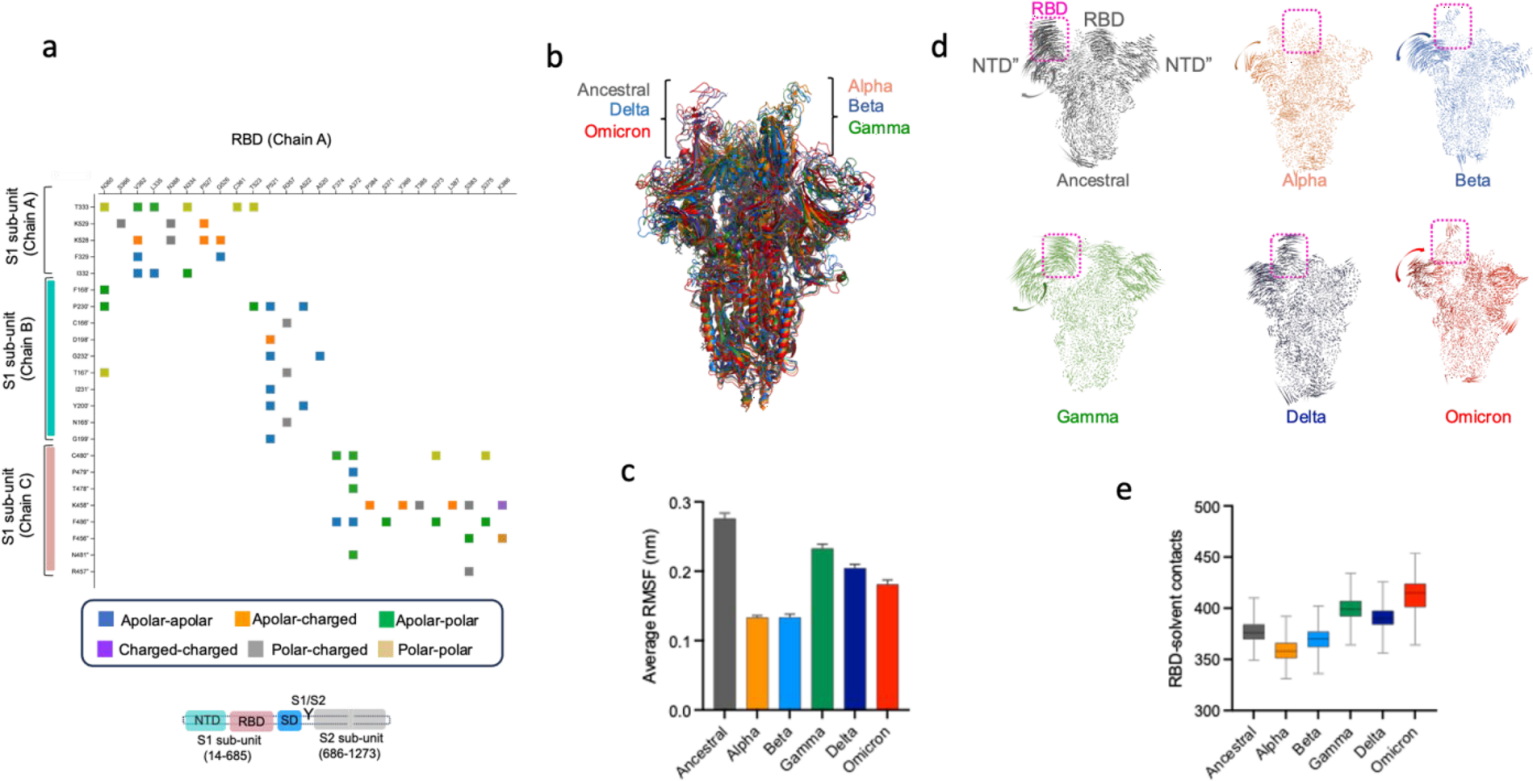
MDS of open-state S protein trimers. (a) Inter-residue contact map formed by a single RBD in one protomer (chain A) with S1, S2 subunits of two neighboring protomers (chains B and C) in the open-state trimeric S protein. The top inset shows the type of inter-residue formed from polar residues: C, H, N, Q, S, T, and W; apolar residues: A, F, G, I, L, V, M, P, and Y; and charged residues: E, D, K, and R. The bottom inset shows S protein domain organization to depict RBD contacts with constitutent domains in neighboring S1, S2 subunits. Unannotated residues represent interdomain (NTD-RBD, RBD-SD) regions. (b) Structural aligment of open-state S protein trimers indicating similar proportions of RBD opening in ancestral SARS-CoV-2 and its VOCs. (c) Cα RMSF analyses of RBD in the open state, averaged over the last 20 ns of the simulation trajectory. (d) Porcupine plots from MDS of open-state S proteins, averaged over the last 20 ns of simulation period. Magnitude and rotational motion of Cα atomic motions are defined by the length and direction of arrows, respectively. (e) Mean solvent contacts of opened RBD obtained from the last 20 ns of simulations. Bars indicate minimum and maximum solvent contact during the simulation period. Color codes for S proteins of ancestral SARS-CoV-2, gray; alpha, orange; beta, light blue; gamma, green; delta, navy blue; and omicron, red.

To assess the kinetic stability of an RBD in its open state, we performed atomistic simulations of fully modeled S protein trimers (using respective cryo-EM structures). Our aim for performing these MDSs was to assess the stability of S protein in an open state, which would have a bearing on engaging with host ACE2 receptors and also recognition by RBD-targeting antibodies. The RBD (chain A) in an open/up state forms a number of polar and nonpolar PPI networks predominantly involving its residues A372, N360, S383, and P521 with NTD′′ and RBD′ of neighboring protomers (chains B and C) (Figure 5a). Alternatively, residues P230′, F456″, K458″, and C480″ (chains B and C) could form the highest intersubunit contacts with opened RBD (chain A) (Figure 5a). To correctly compare kinetic fluctuations, the fully modeled S protein open-state (one-RBD up) structures were structurally aligned, which ensured that RBDs were open to similar proportions in all S proteins (Figure 5b). The kinetic stability was first monitored as a function of Cα-RMSF (root-mean-square fluctuation), averaged over the last 20 ns of the simulation trajectory. The Cα-RMSF analyses showed the highest (∼0.3 nm) fluctuations in ^RBD^ancestral (open state), indicating its highest kinetic mobility (Figure 5c). The RMSFs for ^RBD^alpha and ^RBD^beta were observed to be lowest (∼0.15 nm), indicating their highest kinetic stability followed by ^RBD^gamma, ^RBD^delta, and ^RBD^omicron, where the Cα-RMSF ranged between 0.15 and 0.25 nm. We also performed principal component analysis (PCA) on the dynamic simulation trajectories to isolate collective dominant motions of opened RBD(s) within the respective S protein trimers. In simulations of the open-state ancestral SARS-CoV-2 S protein, we observed large lateral fluctuations in the opened RBD as well as NTD domains of all protomers (Figure 5d). These fluctuations were of relatively lower amplitude in SARS-CoV-2 VOCs: alpha, beta, delta, and omicron. In the alpha, beta, and omicron variants, we observed minimal fluctuations in opened RBD and relatively low amplitude dynamics in the rest of S proteins. Next, the solvent accessibility analyses of S proteins during the later stages of simulation (the last 20 ns) indicated a more solvent-exposed opened ^RBD^omicron/^RBD^gamma (more than 400 average solvent contacts) than ^RBD^ancestral and of other VOCs (less than 375 average solvent contacts) (Figure 5e). This indicated that ^RBD^omicron/^RBD^gamma could engage efficiently with host ACE2 receptors compared to other variants. Taken together, the mutations in S proteins of SARS-CoV-2 VOCs apparently confer more kinetic stability to the RBD in the open state.

### 2.5 Saturation Mutagenesis Predictions

**Figure 6:**
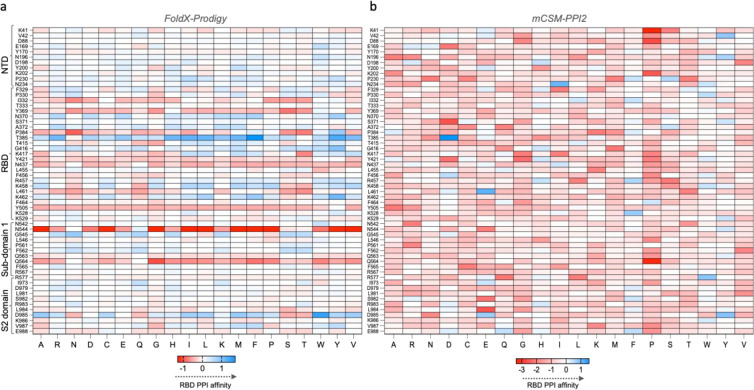
Saturation mutagenesis of residues at the RBD-S1,S2 PPI interface. Saturation mutagenesis of PPI interface residues formed by RBDs in one protomer with S1, S2 subunits of two other protomers in the closed-state S protein, analyzed by (a) FoldX-prodigy and (b) mCSM-PPI2 protocols. Each cell represents a mutation-induced change in the average binding energy from three RBDs in S protein trimer.

To understand the evolutionary selection of mutations at the PPI interface of RBDs and which could its affinity with neighbouring domains, we performed saturation mutagenesis of its interfacial residues. Briefly, 1083 possible mutations were studied based upon 57 amino acids which formed PPI interface for all three RBDs in the closed-state S protein trimer (PDB id:6vxx, cryo-EM model) using FoldX-prodigy and mCSM-PPI2 methods. The resultant matrix from saturation mutagenesis using both methods is shown in Figure 6. In the FoldX-prodigy method, we classified mutations with ΔΔ*G* of less than −0.3 kcal/mol as strongly destabilizing (favoring RBD opening) and more than 0.3 kcal/mol as strongly stabilizing (disfavoring RBD opening), while ΔΔ*G* between −0.3 to 0.3 was classified as moderately stabilizing or destabilizing mutations. We observed 12% and 10.6% strongly stabilizing or destabilizing mutations, respectively (Figure 6a). Quantitatively, mutations at Y369, Y505, and N544 could destabilize while mutations at T385 contributed to the stabilization of RBD interactions with S1, S2 subunits of neighboring protomers. In the mCSM-PPI2 method, mutations of interface residues strongly favored the destabilization of RBD PPI binding affinities (Figure 6b). Quantitatively, 90% of mutations favored destabilization of the PPI interface, with ∼7% of these mutations predicted to cause a reduction in affinity by less than −1.5 kcal/mol. On the other hand, selected mutations such as V42Y, N234I, T385D, L461E, R577W, and D985Y were predicted to increase the PPI affinity of RBDs. In our predictions, we obtained different sets of stabilizing/destabilizing mutations from the FoldX-prodigy and mCSM-PPI2 methods. This may be attributed to methodological differences between these programs in predicting binding affinities at the PPI interface. The FoldX-prodigy method relied on first modeling mutations in proteins using FoldX and then the calculation of binding affinities based upon a linear regression model formed from the contribution of charged, polar, and apolar residues at PPI interface as well as polar and charged noninteracting interfaces. However, the mCSM-PPI2 relies on a graph-based signature approach, which models structural and physicochemical properties of the inter-residue PPI network along with evolutionary information to build a machine-learning-based predictor for assessing the effects of mutations on binding affinity. However, both methods indicated that mutations predominantly in the RBD or subdomain regions can affect the PPI affinity of RBD(s), which can modulate its conformational behavior.

## 3 Discussion

During the evolution of SARS-CoV-2 to its variants of concern, multiple mutations were accumulated in their S proteins (Figure 1c).^25,26^ The high transmission of VOCs was associated with mutations which can increase the host ACE2 affinity or evasion from neutralizing antibodies.^11^ While the predominant effects of these mutations in the VOCs were observed to decrease the ACE2 binding,^27^ the high transmission advantage of SARS-CoV-2 within the population indicates a fine interplay or compensatory epistasis between the host ACE2 affinity and immune evasive potential, which could be regulated by opening transitions in S proteins.^28,29^ In comparative analyses of SARS-CoV-1 and SARS-CoV-2 S proteins, the lower proportions of RBD(s) of SARS-CoV-2 S proteins in the open state were implicated for higher immune evasive capabilities and compensated for lower ACE2 binding (despite the higher binding affinity of isolated RBD(s) compared to that of the SARS-CoV-1 S protein).^4^ The recent emergence of omicron (BA.*) variants also exemplify a high transmission potential within population by exploiting immune-evasive capabilities despite having lower or comparable ACE2 binding affinities compared to those of ancestral or delta SARS-CoV-2.^30,31^ Apparently, as a compensatory mechanism, the accumulation of mutations in SARS-CoV-2 S proteins has resulted in their more human ACE2 accessible, open conformations, which could aid in rapid host receptor scanning and binding (Figure S1). Thus, changes in conformational transitions could present another evolutionary advantage to new variants, which could compensate for ACE2 affinity or antibody neutralization.^4,29^

In the current study, we have employed equilibrium and nonequilibrium atomistic MDS approaches to understand the effects of mutations on the opening behavior of RBD(s) in SARS-CoV-2 VOCs. To gain insight into the propensity of RBD(s) to attain an open conformation or switch between closed and open states, our work focused on protein–protein interaction (PPI) energies of RBD(s), within their closed and open states of trimeric S proteins or during their opening transitions. The main advantage of using a direct analysis of PPI energies of RBD(s) is its sensitivity to point mutations, compared to derived macroscopic parameters such as protein contacts, solvent exposure, changes in folding architecture, etc. The S protein opening was accompanied by an uphill free-energy pathway with a gradual reduction in interdomain protein–protein contacts and thus the PPI energy of transitioning RBDs. Subsequently, substitutions at the PPI interface of the RBDs could modulate the thermodynamic and kinetic free-energy barriers encountered during their opening transitions. The thermodynamic free-energy barrier corresponds only to the free energy of closed and open states and is independent of other barriers, which may be encountered along the opening transition. In our simulations, we observed an approximately 1.5-fold reduction in the thermodynamic free-energy barrier between closed and open states (Δ*G*_closed→open_) for S proteins in VOCs except alpha, where this was similar to ancestral SARS-CoV-2 (Figure 4f). However, the transitions between closed and open states may actually encounter multiple free-energy traps or kinetic free-energy barriers, which would control the rate of RBD transitions. Nonlinear analyses of variations in PPI energy along the RBD opening pathways showed a lower I_50_ (iterations to achieve 50% opening) required by RBDs of SARS-CoV-2 VOCs compared to ^RBD^ancestral. Coupled with the lower slopes of fitted curves, the results indicate apparent faster transitions in the VOCs (Figure 4e–f). The flattened slopes observed in VOCs may also reflect a lower kinetic free-energy barrier in transitioning the uphill RBD opening pathways. While in our analyses we could not determine kinetic traps along the reaction coordinate, the less-steep free-energy transitions (especially for ^RBD^omicron) were congruent with rigid-body domain analyses, where RBD(s) opening in S proteins of SARS-CoV-2 VOCs were accompanied by a lower amount of closure/opening motion and angular rotation compared to ^RBD^ancestral (Figure 4a,b). For ancestral SARS-CoV-2 and B.1 variant S protein opening kinetics, Díaz-Salinas et al. have also proposed that mutations such as D614G can reduce Δ*G*_closed→open_, stabilizing the open-state conformations of RBD.^6^ Also, they have shown that these transitions are independent of ACE2 binding, which otherwise stabilizes the RBD-up state and reduces the transition to the RBD-down conformation. The closure/opening motion for ^RBD^omicron and ^RBD^delta was similar to RBD(s) of SARS-CoV-1 (∼21%), whose opening also proceeded through a relatively flat free-energy pathway compared to that of ^RBD^ancestral^5^ and shows higher propensity of its RBD(s) in the open/up state.^4^ From a thermodynamics standpoint, a less-steep free-energy landscape (e.g., of ^RBD^omicron) could also apparently increase the configurational entropy of S protein, which would favor access of S protein to available ACE2 conformations.^5,32^ These swift transitions coupled with the high atomic fluctuations observed for closed- and open-state S protein trimers of omicron and gamma variants correlate with cryo-EM reports indicating highly mobile RBD(s), which predominantly occupied up states (Figure S1).^10,12^ Interestingly, the effects of mutations in S protein were also reflected in kinetic fluctuations of RBD in the open/up state (Figure 5). Compared to ^RBD^ancestral, RBDs (open/up state) in VOCs showed higher kinetic stability, which was consistent with recent single-molecule Förster resonance energy (FRET) experiments where enhanced kinetic stabilization of open-state S protein was observed.^33^ The stabilization effects were also extended to a more solvent-exposed open-RBD in S proteins of omicron and gamma variants. From the previous knowledge on the role of the structural transition in S proteins, such effects can impact SARS-CoV-2 host tropism in multiple ways. The substitutions which translate/stabilize RBD in the up state could facilitate interactions with host ACE2 receptors^4^ or immune recognition by neutralizing antibodies, which target ACE2 binding-RBM in open states.^5,11,17^ Alternatively, the stabilization of RBD in the up state (in omicron, Figure 5c,d) could also impair neutralization by antibodies such as S304 which specifically targets the closed conformation of S protein.^34^ Thus, the RBD mutations could have differential roles in ACE2 binding and immune recognition.^29,35^ Based upon our observations, we hypothesize that the enhanced kinetic stability may have a role in increased residence times of RBD in up/open states for an optimized interaction with ACE2 receptors. On the other hand, a weak thermodynamic free-energy barrier (Δ*G*_closed→open_) can promote rapid switching to closed states, which along with antibody-escaping mutations can confer higher immune evasive capabilities. Further insights from protein–protein contact maps in closed-state S protein and in-silico saturation mutagenesis suggest that mutations were not randomly selected over the course of evolution. The high protein–protein contact frequency of residues K41, E169, Y200, F329, I332, R355, Y369, N370, S371, A372, T385, K386, D389, L390, T415, K458, L461, K462, H519, A520, P521, K528, N544, Q564, R983, L984, and D985 between RBD-S1, S2 subunits coupled with the higher predicted stabilization/destabilization tendency of some residues indicates their role in maintaining the integrity of RBDs within S protein (Figures 2a and 6a,b). Interestingly, the predicted highly stabilizing/destabilizing mutations had significantly low prevalence (present in less than 1% of sequenced SARS-CoV-2 isolates)^36^ compared to highly prevalent (more than 80%) RBD interface mutations such as S982A, K417N, K417T, S371L, S373P, S375F, Y505H, and L981F, which showed moderate destabilizing effects on RBD affinity (−0.2 to −0.3 kcal/mol in FoldX-prodigy and −0.3 to −1 kcal/mol in mCSM-PPI2). The low prevalence of these highly destabilizing/stabilizing mutations indicates that the evolution of S protein apparently balances efficient host–receptor binding and overall S protein stability. However, in the future, these mutations may occur along with other S protein substitutions to maintain compensatory epistasis. While we predicted the impact of single mutation in affecting RBD(s) PPI affinity, the accumulation of multiple mutations at the interface such as in BA.* lineages could impact structural dynamics in a distinct manner. Our study might also explain that despite accumulating multiple mutations in RBD which can decrease ACE2 affinity,^27^ collectively they can modulate conformational transitions in S protein for efficient host–receptor scanning and binding. It may also be worth noting that environmental variations or substitutions in ACE2 can also modulate its conformational freedom and affect its binding behavior with RBDs. In this direction, using extensive MDS, Lecot et al. have demonstrated that the adsorption of ACE2 on specific silane monolayers could increase its binding affinity with RBD.^37^ Changes in the transition behavior of RBD(s) could also affect currently proposed or future antiviral strategies which aim to stabilize the closed conformation of S proteins such as observed for highly conserved free fatty acid binding pockets formed between two RBDs.^38,39^ On the other hand, understanding the free-energy pathways of transitions might aid in the design of molecules, which can effectively target druggable pockets in intermediate structures rather than closed/open states.

## 4 Conclusions

The mapping of conformational transition pathways can aid in our mechanistic understanding of SARS-CoV-2 spike (S) proteins. We observe that the accumulation of nonsurface mutations can not only regulate host ACE2 binding behavior or evasion from neutralizing antibodies but also modulate RBD opening pathways in S protein. The conformational transitions had also been previously implicated in regulating S protein and ACE2 binding kinetics despite differences in the individual binding affinity of RBD. Upon lowering the free-energy barrier (Δ*G*_closed→open_), the new SARS-CoV-2 variants can regulate open or closed states of RBD, which can have a bearing on immune evasive capabilities. Overall, our study provides new insights into the adaptation of SARS-CoV-2 variants, to the main transmission advantage within a population.

### 4.1 Limitations of the Study

The SARS-CoV-2 S protein is heavily glycosylated and can potentially modulate RBD opening dynamics.^40^ In our study, we have used nonglycosylated forms to exclude the contributions of differential glycosylation patterns from intrinsic S protein dynamics, in modulating the opening pathways. While our study focuses on direct contributions of mutations at the PPI interface, the modulation through allosteric networks can also impact S protein opening dynamics. Despite these limitations, our study highlights the possible role of intrinsic S protein mutations in its opening dynamics, and the selection of these mutations could form the driving force for the evolution of SARS-CoV-2.

## 5 Methodology

### 5.1 Retrieval of Cryo-EM Models, Sequence Analysis, and Equilibrium MDS

The cryo-EM structures for the analysis of closed-state or open-state S protein trimers were retrieved from rcsb.org/. In all structures, missing loop regions in cryo-EM structures of S proteins were homology modeled using the Swiss model with respective structures as templates.^19^ Sequences for multiple sequence alignment were retrieved from uniprot.org/ and performed using Clustal Omega (ebi.ac.uk/Tools/msa/clustalo/). Angular rotation and amount of RBD closure in its rigid body domain motion analysis was done using DynDom3D.^41^ RBD motion was analysed using standard parameters of 4 Å total grid size with a 0.6 occupancy factor. Atomistic MDS (1 μs) of all closed- and open-state SARS-CoV-2 S protein trimers (ancestral and VOCs) was performed with GROMACS v2018.1 and the gromos54a7 force field.^42^ All His residues in S proteins were protonated using the GROMACS tool “gmx pdb2gmx” on either the N_δ1_ or N_ε2_ atom (neutral histidines) to maintain an optimal H-bond conformation. The proteins were placed in a cubical box with 10 nm spacing from the box edges and solvated at 0.15 M NaCl (to mimic physiological salt concentration), along with appropriate number of added counterions to maintain system electroneutrality. The system was then energy minimized using a steepest-descent protocol followed by NVT (constant number of particles, system volume, and temperature) and NPT (constant number of particles, system pressure, and temperature) equilibration for 500 ps. In our simulations, short-range electrostatic and van der Waals interaction cutoffs were kept at 1 nm while long-range electrostatic interactions were treated with particle-mesh Ewald^43^ summation, with a 0.14 nm grid spacing. Bond lengths were constrained with the LINCS^44^ algorithm. System temperature (300 K) was controlled using the Velocity rescaling thermostat,^45^ and its pressure was controlled using a Parrinello–Rahman barostat^46^ (1 bar reference pressure) with a compressibility of 4.5 × 10^–5^ bar and an isotropic scaling scheme. Simulation output trajectories were analyzed using in-built gromacs tools. We further performed principal component analyses of simulation trajectories of open-state S proteins averaged over the last 20 ns to collect large-amplitude dynamics. Correspondingly, mass-weighted covariance matrices were generated for Cα atoms of S protein trimers, and output trajectories were projected onto the first eigenvector. The Cα fluctuations were derived from extremer projections and plotted using PyMol (The PyMOL Molecular Graphics System, version 2.0, Schrödinger, LLC). Plots were generated using GraphPad Prism v9.3.1 (GraphPad Software, San Diego, CA USA, www.graphpad.com).

### 5.2 Nonequilibrium Opening of S Protein RBD(s)

The full pathway for the conformational transition of S protein from the closed to open state was mapped by stitching elastic network model (ENM) calculations with atomistic MDS. The ENM method (iMorph v1.44 suite)^47^ was based upon calculations of internal coordinates (defined by backbone dihedral (ϕ, ψ) angles) and can be defined by the harmonic potential as
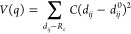
where *d_ij_* and *d*_*ij*_^0^ represent the internodal (*i* and *j*) or interatomic distance final and initial structure, *C* is the spring constant the between *i*–*j* pair, and *R*_c_ is the cutoff radius of 7.0–8.0 Å.

The morphing transitions were based on iterative deformations from the initial structure, and the resulting displacements were selected on the basis of their eigenvalues and merged using random amplitudes. The new conformations are accepted if the backbone RMSD decreases or converges toward the final structure; otherwise, new modes are selected for displacement. In the current study, closed-state S protein trimer backbone atoms were chosen for morphing transitions, with a cutoff RMSD of 0.3 nm from respective open states. Intermediate structures (*n* = 75) sampled through iMorph were subjected to independent atomistic MDS using different initial velocities with gromos54a7 force fields and stitched to achieve an opening transition in 75 ns. In total, 13 μs of data was generated and used for comparative analyses of S proteins. The nonlinear curve fitting analyses of conformational transition data were completed using the Boltzmann equation, implemented in GraphPad Prism v9.3.1 (GraphPad Software, San Diego, CA, USA, www.graphpad.com).
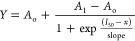
where *A*_1_ and *A*_o_ are the top and bottom of the curve, respectively, and *I*_50_ is the half-point between *A*_1_ and *A*_o_.

### 5.3 Predicting the Effects of Mutations on PPI Affinity

To predict the effect of point mutations on the PPI affinity of RBDs with neighboring S1, S2 subunits, the mutant spike proteins were modeled using FoldX v4.0^20^ suite locally with the number of runs set to 5 to achieve convergence. Binding free energy analyses of RBDs were carried through contact-based calculations implemented in the prodigy tool.^18^ The prodigy program for calculating PPI energies is based upon the linear regression treatment of both interfacial protein–protein contacts and the properties of noninteracting surfaces. The predicted binding free energy (Δ*G*_predicted_) correlates closely to the magnitude of experimental binding affinities and can be expressed as
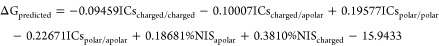
where IC and NIS are the interfacial contacts and noninteracting surfaces, respectively.

The effect of point mutations on the PPI affinity of RBDs was also analyzed using mCSM-PPI2,^21^ which uses a graph-based signature approach to assess the effects of mutations on the intermolecular contact network.

Changes in binding affinity upon mutations could be represented as
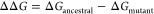


### 5.4 Saturation Mutagenesis Predictions

Saturation mutagenesis predictions were made using a combined FoldX-Prodigy and FoldX-mCSM-PPI2 method. First, the PPI interface residues formed among all three RBDs and neighboring S1, S2 subunits were determined from prodigy^18^ analysis of the cryo-EM structure of ancestral SARS-CoV-2 S protein (PDB id: 6vxx). The selected interface residues were used to prepare the mutant library using the FoldX mutation engine.^20^ The FoldX modeled mutant structures were then subjected to Prodigy and mCSM-PPI2-based analyses to obtain changes in the binding affinity upon acquiring mutations.

## Supporting Information Available

Supplements to main text and figures and list of PDB codes for preliminary analysis of closed-state S protein trimers (PDF)
