# Supplementary material for: Spike Protein Mutation-Induced Changes in the Kinetic and Thermodynamic Behavior of Its Receptor Binding Domains Explain Their Higher Propensity to Attain Open States in SARS-CoV-2 Variants of Concern
Source: ACS Cent Sci. 2023 Sep 21;9(10):1894–904. doi: 10.1021/acscentsci.3c00810 (PMC10604015; doi:10.1021/acscentsci.3c00810)
Supplement: Supplementary file 1 — oc3c00810_si_001.pdf [file oc3c00810_si_001.pdf]

Supplementary Information

Spike protein mutations induced changes in kinetic and thermodynamic behaviour of its receptor binding domains explains their higher propensity to attain open states in SARS-CoV-2 variants of concern

Jasdeep Singh[1*#], Shubham Vashishtha[2*], Bishwajit Kundu[2#]

[1] Department of Chemistry and Biochemistry, University of Denver, Denver-80208, USA;

[2] Kusuma School of Biological Sciences, Indian Institute of Technology-Delhi, New Delhi-110016, India

[#]Corresponding authors

Dr. Jasdeep Singh
email: jasdeep002@gmail.com, jasdeep.singh@du.edu

Prof. Bishwajit Kundu
email: bkundu@iitd.ac.in

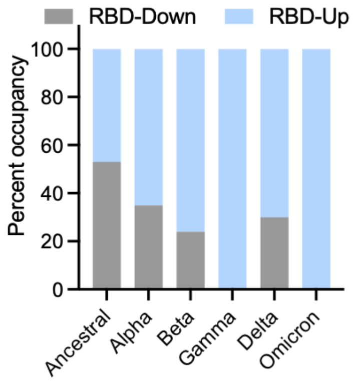

Figure S1. Stacked plots of occupancy of RBD(s) in “down/closed” (grey) and “up/open” (blue) states, adapted from cryo-EM structure data [1, 10, 15-17].

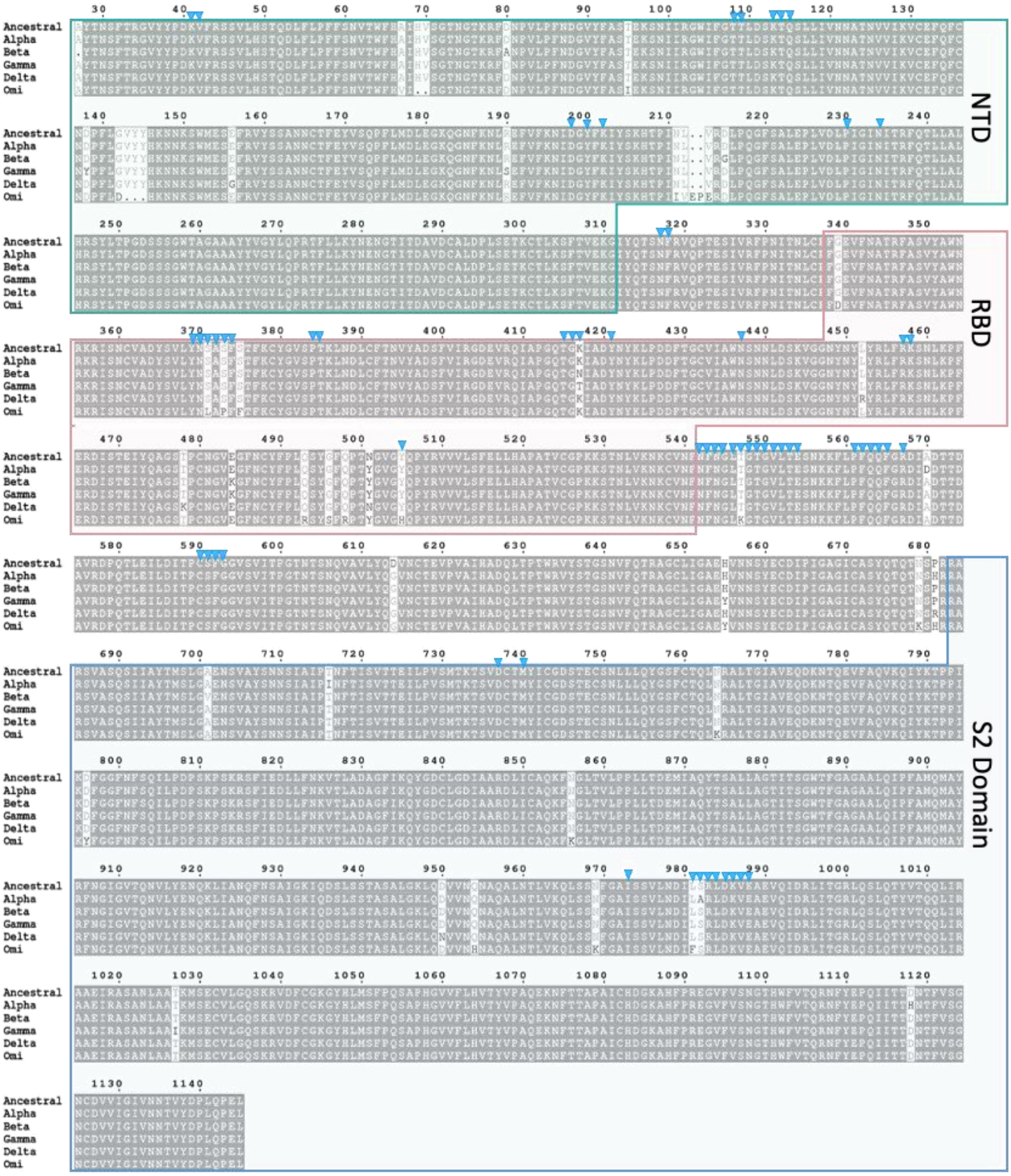

Figure S2. Multiple sequence alignment of ancestral SARS-CoV-2 and its VOCs: Alpha, Beta, Gamma, Delta, and Omicron. Blue triangles indicate interfacial residues between a RBD of one protomer and the adjacent S1 and S2 domains of other two protomers.

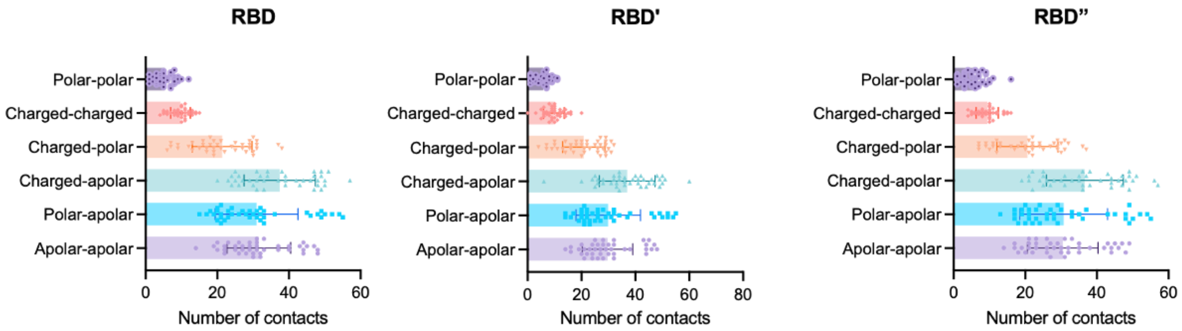

Figure S3. Qualitative and quantitative assessment of protein-protein contacts formed by three RBDs with S1 and S2 domains of other two protomers in S protein structures (Table S1) of ancestral SARS-CoV-2. The type of protein-protein contacts was obtained through prodigy-based analyses of individual structures.

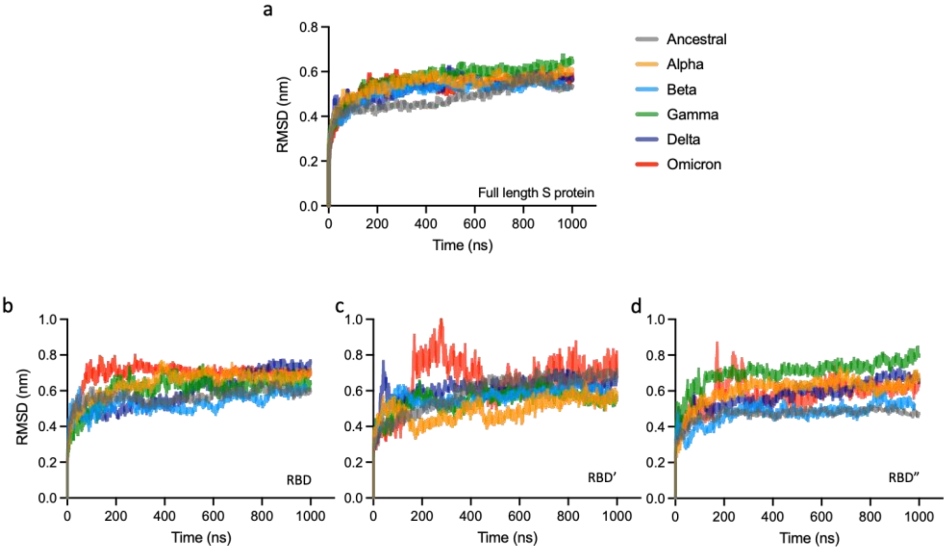

Figure S4. Variations in Cα-RMSD of closed state S protein trimers. (a) Variations in Cα-RMSD of S proteins by aligning to their respective starting structures. (b-d) Variations in Cα-RMSD of three RBDs by aligning to starting structures of respective entire S proteins.

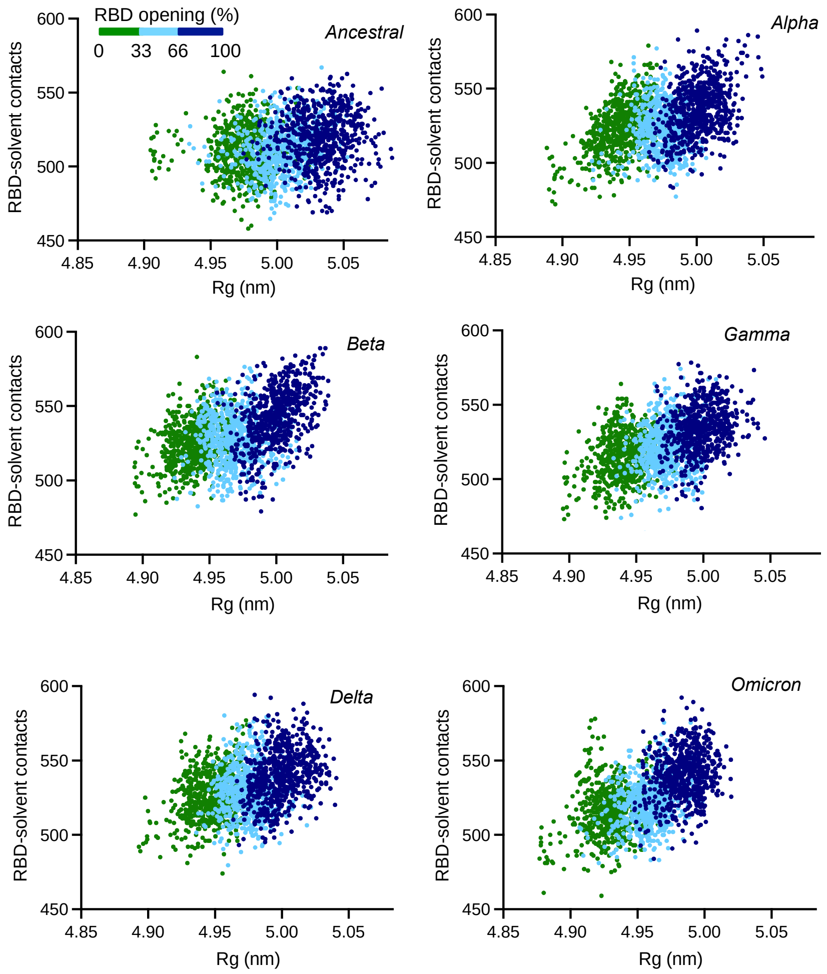

Figure S5. Two dimesional plots (gyration radius (Rg) vs solvent contacts) for RBD opening in S proteins, mapped from MD simulations of structures obtained from individual morphing runs.

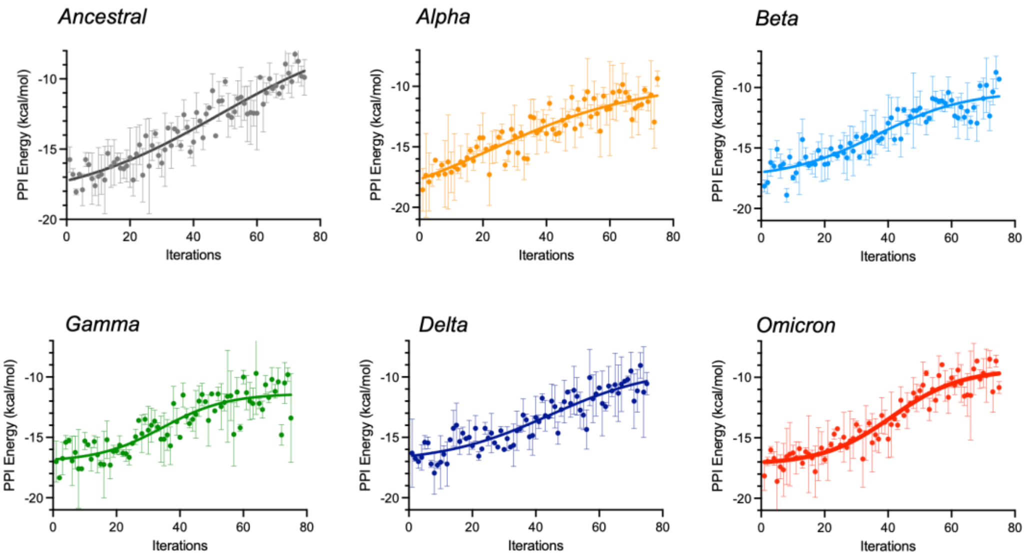

Figure S6. Variations in PPI energy of single RBD with its adjacent domains along the opening pathway of S proteins of ancestral SARS-CoV-2 and its VOCs. Solid lines depict non-linnear curve fitting of RBD PPI energy along the opening pathways. Bars indicate standard error of mean PPI energy from two independent simulations.

Table S1. List of PDB codes used in analysis of protein-protein contacts formed by RBD with neighbouring S1, S2 domains in closed state S protein trimers.

6x29,6xf5,6zb4,6zb5,6zge,7ddd,7df3,7dwy,7fb0,7jji,7kdg,7mkl,7mw2,7nda,7ndc,7qur,7qus,7tla,7tlb,7wgv,7z3z,7jwy,6zp0,5xlr,6x79,6vxx,6x6p,7cab,6xr8,7nt9,7n1u,7n1t,6zgi,6zwv,7krs,7n1u,6zgi,7krq,7krr,7y42
